# What Really Drives Economic Growth in Sub-Saharan Africa? Evidence from the Lasso Regularization and Inferential Techniques

**DOI:** 10.1007/s13132-022-01055-1

**Published:** 2022-10-21

**Authors:** Isaac K. Ofori, Camara K. Obeng, Simplice A. Asongu

**Affiliations:** 1 Department of Economics, University of Insubria, Via Monte Generoso, 71, 21100 Varese, Italy; 2 The Harriet Tubman Institute, York University, 4700 Keele Street, Toronto, On M3J 1P3 Canada; 3 Economic Policy Division, Faculty of Economics and Business Administration, Friedrich Schiller University of Jena, Jena, Germany; 4 Department of Economic Studies, School of Economics, University of Cape Coast, Cape Coast, Ghana; 5 School of Economics, University of Johannesburg, Johannesburg, South Africa

**Keywords:** Economic growth, Elasticnet, Lasso, Machine learning, Partialing-out IV regression, Sub-Saharan Africa

## Abstract

The question of what really drives economic growth in sub-Saharan Africa (SSA) has been debated for many decades now. However, there is still a lack of clarity on the variables crucial for driving growth as prior contributions have been executed at the backdrop of preferential selection of covariates in the midst of several potential drivers of economic growth. The main challenge with such contributions is that even tenuous variables may be deemed influential under some model specifications and assumptions. To address this and inform policy appropriately, we train algorithms for four machine learning regularization techniques— *the Standard lasso*, *the Adaptive lasso*, *the minimum Schwarz Bayesian information criterion lasso*, and *the ElasticNet—* to study patterns in a dataset containing 113 covariates and identify the key variables affecting growth in SSA. We find that only 7 covariates are key for driving growth in SSA. The estimates of these variables are provided by running the lasso inferential techniques of *double-selection linear regression*, *partialing-out lasso linear regression*, and *partialing-out lasso instrumental variable regression*. Policy recommendations are also provided in line with the AfCFTA and the green growth agenda of the region.

## Introduction

The debate on the sources of growth continues to generate attention in the political and academic landscapes due to its relevance for policy formulations on welfare, international competition, and economic management. From the saving-oriented (Domar, 1947; Harrod, 1939) and technical progress neoclassical theories of economic growth (Solow, 1956) to the imperfect market-augmented endogenous growth theories of Romer (1990), Aghion and Howitt (1990), and Grossman and Helpman (1991), economists are still exploring which variables matter for growth. The need to identify the key drivers of economic growth has even become crucial than ever following the emergence of the coronavirus pandemic (IMF, 2020; World Bank, 2020). For developing economies such as those in sub-Saharan Africa (SSA), knowledge on the key drivers of economic growth is a great step in formulating and implementing policies to foster, sustain, and share growth. Additionally, identifying the key drivers of economic growth would be a giant breakthrough on the parts of policymakers and developing partners in mapping out growth strategies in line with the *green growth*^1^* agenda* of the region.

A plethora of prior contributions identifies covariates such as trade openness, foreign direct investment, and innovation (Agbloyor et al., 2014; Sakyi et al., 2015), financial development (Opoku et al., 2019; Peprah et al., 2019), macroeconomic management (Alagidede & Ibrahim, 2017), institutional quality (Berhane, 2018; Chakamera, 2018), human capital (Anyanwu, 2014; Gyimah-Brempong et al., 2006), and ICT (Adeleye & Eboagu, 2019; Asongu & Odhiambo, 2019) as drivers of economic growth in SSA. A conspicuous lacuna in the extant scholarship, however, is that, all these variables deemed crucial for economic growth are selected based on the researcher’s discretion even in large dataset regression problems. The concern with preferential selection of covariates is that, even *tenuous* drivers of growth may be deemed highly influential under certain assumptions, model specification, or estimation techniques. Another challenge is that, the preferential selection of covariates in the midst of several potential determinants of growth partly contributes to the inconclusive results in big data regression problems. Addressing this challenge and thus informing policy appropriately can be through the application of machine learning^2^ (artificial intelligence) techniques for regularization, and inference (see Tibshirani, 1996; Zou & Hastie, 2005; Zou, 2006). Indeed, machine learning techniques have been applied in various fields, for example, in health (see Mateen et al., 2020; Doupe et al., 2019; Beam & Kohane, 2018), transportation (Bhavsar et al., 2017; Tizghadam et al., 2019), games and psychology (Sandeep et al., 2020; De Almeida-Rocha & Duarte, 2019; Luxton, 2016), and finance (Bredt, 2019; Bazarbash, 2019; Akbari et al., 2021).

Despite the rise in the application of machine learning techniques in several fields, rigorous empirical works exploring its applicability and power in selecting variables crucial for economic growth in SSA are hard to find. This fundamentally forms the contribution of this paper. The first objective, therefore, is to train several machine learning algorithms to identify the main drivers of economic growth in SSA. The second objective is to provide reliable estimates and confidence intervals for these main determinants of economic growth, taking into consideration possible endogeneity, multicollinearity, and modeling complexities. To the best of our knowledge, this study is the first of its kind in SSA to apply machine learning techniques in selecting the main drivers of economic growth. Particularly, following renewed efforts to achieve, sustain, and share growth gains in line with implementation of the African Continental Free Trade Area (AfCFTA) and the institution of the African Agenda 2063, our results could prove crucial to the course by aiding the planning, modeling, and the targeting of growth.

Our choice of the study area is informed by a number of factors. First, as Kaufman et al. (2010) note, SSA countries are fundamentally common in terms of institutions. Despite lags in several facets of governance such as the rule of law, regulatory quality, and corruption-control, the quality of these indicators, as the World Governance Indicators suggest, is rising steadily across the region. However, macroeconomic challenges relating to inflation, exchange rate fluctuations, macroeconomic bailouts, and geopolitical fragilities are common among countries in the region. Second, SSA countries are remarkably similar in terms of structural or real sector setting (OECD/ILO, 2019; UNCTAD, 2021; World Bank, 2021a). For instance, most of the region’s active workforce is employed in the agricultural sector and are more susceptible to political, financial, and trade shocks. Also worth mentioning is the common goal of SSA countries in using economic integration^3^ as a vehicle to spur industrialization, growth, poverty alleviation, and equitable income distribution. Another peculiarity is the low industrial output but fast rising service sector, providing policymakers with opportunities to leapfrog classical development processes (IMF 2020). Third, as noted by the African Development Bank (2018), countries in the region are markedly common in infrastructural development. Particularly, SSA countries report sharp deficits in digital and physical infrastructure such as ICT, electricity, transportation, as well as water and sanitation compared to their North African counterparts. Finally, countries in SSA are substantially similar in terms of growth trajectories, level of development, and lingering concerns of inability to build sustained growth momentum.

The rest of the paper is organized as follows: the next section presents a brief survey of economics-related studies applying machine learning. The data and empirical models are also presented in Sect. 3. The results and discussions are presented in Sect. 4 while Sect. 5 concludes with some policy recommendations.

## Literature Survey on Empirical Works Using Machine Learning

The literature on economic growth is vast and an attempt to present all of them will be a daunting one. Therefore, attention is paid to the recent advances and applications of machine learning regularization techniques in the area of growth and development. For instance, this study is similar to Schneider and Wagner (2012) who focus solely on the lasso (least absolute shrinkage and selection operator) in determining the key drivers of growth in the NUTS2 region^4^ of the European Union over the period 1995–2005. The results indicate that covariates such as initial GDP per capita, human capital, and initial unemployment rate matter for economic growth.

Similarly, in identifying which income distribution measure matter for development outcomes, Dutt and Tsetlin (2016) applied the Elasticnet and the lasso techniques to select from 37 potential covariates of development. The authors find that the poverty headcount indicator matters most in predicting three development outcomes (i.e., per capita income, schooling, and institutional quality). A similar work is Tkacz (2001), which, in forecasting Canadian GDP growth, applied the neural network algorithms. The study finds that, relative to traditional methods such as the linear and univariate forecasting methods, neural network techniques yield lower forecast errors on annual growth rate. The author goes further to indicate that neural techniques perform better in forecasting long-term growth than short-term growth. Further, Richardson et al. (2021) explore the power of several machine learning techniques^5^ relative to classical methods in forecasting real GDP growth in New Zealand. The authors find that machine learning algorithms outperform classical statistical methods in prediction. Jung et al. (2018) also employ machine learning algorithms of lasso, ridge, Elasticnet, neural networks, and super learner to examine the GDP growth of the G7 countries. The authors provide strong evidence to conclude that machine learning algorithms outperformed standard prediction techniques.

**Fig. 1:**
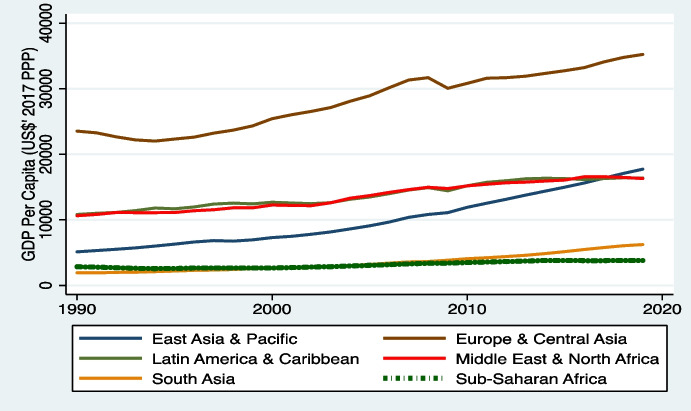
Trend of GDP per capita across regions, 1990–2019. Source: authors’ construct using data from World Development Indicators

In the case of SSA, however, the literature shows that researchers have not explored how relevant these techniques can be in aiding policymakers plan and target growth. The results we provide could prove invaluable in helping policymakers turn around the slow growth (real GDP per capita) trajectories of the SSA as presented in Figs. 1 and 8 in the Appendix.

### Literature Survey on Drivers of Economic Growth Based on Traditional Techniques

In this section, we present a survey of the literature on the effects of several covariates included in this study on economic growth. Using a dataset on 21 SSA countries for the period 2000–2014, Ngongang (2015) employed the dynamic GMM technique to examine the relationship between financial development and economic growth. The author finds a significant positive relationship between the variables. In the same way, Ibrahim and Alagidede (2018) use a panel dataset spanning 1980–2014 for 29 SSA countries to examine the conditional and unconditional effects of financial development in economic growth. The results suggest that while financial development has a positive impact on economic growth, the joint effect of financial development and investment is rather remarkable. Kodongo and Ojah (2016) also explore the link between infrastructure and economic development in SSA countries. The results, which are based on system GMM estimator and a dataset on 45 SSA countries for the period 2000–2011, show that relative to middle-income countries, infrastructure plays a salient role in the economic development of least developed countries.

Omoteso and Mobolaji (2014) also apply the panel fixed effect, random effect, and the maximum likelihood estimation techniques to test the linear relationship between governance and economic growth in some selected SSA countries for the period 2002 to 2009. The authors find strong evidence to conclude that while political stability and regulatory quality enhance growth, government effectiveness adversely affect economic growth. Using a panel of 27 countries in SSA, Kebede and Takyi (2017) also employed the panel causality and system GMM estimation techniques to examine the relationship between institutional quality and economic growth. While the authors find a unidirectional causality from economic growth to institutional quality, the reverse does not hold. The results further show that institutional quality, trade openness, financial development, and debt positively affect economic growth.

In exploring the link between government expenditure and economic growth, Olaoye et al. (2020) apply the system GMM and the Driscoll and Kraay estimator to examine the asymmetrical phenomenon in government spending and growth relationship in 15 ECOWAS countries. Aside from confirming the asymmetric link between government spending and economic growth, the authors find evidence of an inverted U-shaped connection between government spending and economic growth. Also, Adams and Opoku (2015) investigate the effect of FDI on economic growth using a panel of 22 SSA for the period 1980–2011. The authors find evidence from the GMM estimator to show that although unconditionally FDI does not drive economic growth, the joint effect of FDI and regulations is positive and statistically significantly. Adams et al. (2016) also examine the link between energy consumption and economic growth, and the modulating role of democracy using a panel data of 16 SSA countries from 1971 to 2013. The study provides evidence from the panel vector autoregressive model to show that energy consumption enhances economic growth in the region. The study further finds that the joint effect of democracy and energy consumption on economic growth is positive and significant.

In addition, Adams and Klobodu (2016) assess the effect of remittances and regime change on economic growth for 33 SSA counties over the period 1970–2012. Their results from the system GMM estimation technique show that while remittances do not significantly affect growth, regime change suppresses growth. The study concludes that the growth-enhancing effect of remittances is amplified in the presence of a democratic and stable government. Appiah-Otoo and Song (2021) also use a panel of 123 countries composed of 45 high-income countries, 58 middle-income countries, and 20 low-income countries for the period 2002–2017 to examine the impact of ICT on economic growth. The authors provide strong evidence that the effect of ICT diffusion on growth across rich and poor countries is significantly different and that poor countries tend to gain more from the ICT innovations. Employing a panel dataset on 20 African countries, Akadiri and Akadiri (2018) applied the fixed effect estimator to test the relationship between growth and income inequality, on the one hand, and the pathway through which growth determinants influence income inequality for the period 1991 to 2015. The study finds evidence of positive long-run relationship between income inequality and growth. The study further reveals that population growth, mortality rate, government consumption expenditure, and foreign direct investment are principal determinants of the long-run growth and income inequality in the sampled countries.

In the same vein, Mavikela et al. (2019) examined the effect of inflation on economic growth for South Africa and Ghana with data over the period 2001 to 2016. Evidence from the quantile regression shows that while high inflation is positively related with growth in Ghana, it is the opposite in the case of South Africa. The study further shows an adverse effect of inflation at all threshold levels on growth in the post 2008/2009 global financial crisis.

## Data and Methodology

### Data

The study employed a large balanced panel spanning 1980–2019 for the analysis. The study sampled 42 SSA countries^6^ on grounds of data availability. The outcome variable, economic growth, is the annual real GDP growth rate and is drawn from the World Development Indicators (World Bank, 2021b). Data on 113 potential drivers of growth are considered based on the extant scholarship on economic growth. Taking into consideration the real sector of the economies under consideration, variables such as vulnerable employment, inflation, and self-employment are considered (Bittencourt et al., 2015; Barro, 2013). Likewise, we include variables such as trade openness, and tariff considering the rise in economic globalization of SSA following the implementation of the AfCFTA and the projected rise in FDI inflow to the region in 2022 (UNCTAD, 2021; OECD/ACET, 2020). The essence of economic integration for growth in marginalized settings like SSA rests in the classical trade argument that it can foster social progress and the contemporary view that trade is essential for innovation diffusion, technological transfer, global value chain participation and export diversification (Asongu & Odhiambo, 2019; Asongu & Nwchukwu, 2016; Sakyi et al., 2015; Adams & Opoku., 2015).

Variables such as financial development and infrastructure are also considered due to their contribution to growth through resource allocation and the facilitation of economic activities (Koomson et al., 2020; Opoku et al., 2019; Peprah et al., 2019, African Development Bank*,* 2018). We source data on financial development from the World Bank’s Global Financial Development Database (Čihák et al., 2013) and the International Monetary Fund’s financial development index (Svirydzenka, 2016). Also, the study includes welfare variables of poverty and inequality due to their prevalence in the selected countries despite gains chalked in recent years and the fact that such developments waste human capital, consequently dragging down growth.

Data on poverty and inequality are sourced from the World Bank’s Poverty and Equity Database, and the Global Consumption and Poverty Project (Lahoti et al., 2016), while that of globalisation^7^ is drawn from the Konjunkturforschungsstelle (KOF) index (Gygli et al., 2019). Per empirical evidence on the contribution of institutions and policy to growth, we consider country policy and institutional scores on macroeconomic management, trade policy, social protection, social inclusion, and financial sector management (Akobeng, 2016; Anyanwu, 2003; Asongu & Gupta, 2015; Asongu & Nwachukwu, 2017; Fosu, 2012). Also, we consider ICT skills, access, and usage given the momentous rise in the digital infrastructure of the region (Appiah-Otoo & Song, 2021; Tchamyou et al., 2019; Adeleye et al*.,* 2019). The definitions and sources of all the variables are reported in Table 1.

**Table 1:** Variable definition and data sources

Variable	Description	Source
women	Proportion of seats held by women in national parliament (%)	WDI
Women_business	Women businesses and law index score (scale 1–100)	WDI
wagessalary	Wage and salaried workers, total (% of total employment)	WDI
vul_tot	Vulnerable employment is contributing family workers and own-account workers as a percentage of total employment	WDI
lossesdue_power	Productivity losses due to power	WDI
urban_pop	Urban population growth (annual %)	WDI
unempl	Unemployment, total (% of total labor force)	WDI
trade	Trade is the sum of exports and imports of goods and services measured as a share of gross domestic product	WDI
trade_tax	Taxes on international trade include import duties, export duties, profits of export or import monopolies, exchange profits, and exchange taxes	WDI
taxrev	Tax revenue (% of GDP)	WDI
tariffwm	Tariff rate, applied, weighted mean, all products (%)	WDI
self_employ	Self-employed, total (% of total employment)	WDI
rur_popgrof	Rural population growth (annual %)	WDI
rd	Research and development expenditure (% of GDP)	WDI
rer	Real effective exchange rate index (2010 = 100)	WDI
HIV_preva	Prevalence of HIV, total (% of population ages 15–49)	WDI
prenatal	Pregnant women receiving prenatal care (%)	WDI
povert_hc	Poverty headcount ratio at national poverty lines (% of population)	PED
povertyhc_mid	Poverty headcount ratio at $3.20 a day (2011 PPP) (% of population)	PED
povertyhc_low	Poverty headcount ratio at $1.90 a day (2011 PPP) (% of population)	PED
povmidd	Poverty gap at $3.20 a day (2011 PPP) (%)	PED
povint	Poverty gap at $1.90 a day (2011 PPP) (%)	PED
urbanization	Urban population (% of total population)	WDI
popgrof	Population growth (annual %)	WDI
finan_insti	Deposit money banks' assets to GDP (%)	WDI
sanitation_pop	People using at least basic sanitation services (% of population)	WDI
opendefeca_pop	People practicing open defecation (% of population)	WDI
exr	Nominal exchange rate (US dollar)	WDI
unfpa_aid	Net official flows from UN agencies, UNFPA (current US$)	WDI
unicef_aid	Net official flows from UN agencies, UNICEF (current US$)	WDI
undp_aid	Net official flows from UN agencies, UNDP (current US$)	WDI
noda	Net Official Development Assistance received (% of GNI)	WDI
netmigration	Five-year estimates on net migration (immigrants less emigrants)	WDI
mortality_5yrs	Number of under-five deaths	WDI
manuf_VA	Manufacturing, value added (annual % growth)	WDI
logisticqua_overal	Logistics performance index: overall (1 = low to 5 = high)	WDI
logisticqua_TT	Quality of trade and transport-related infrastructure (1 = low to 5 = high)	WDI
logisticqua_ship	Logistics performance index: frequency with which shipments reach consignee within scheduled or expected time (1 = low to 5 = high)	WDI
logisticqua_custom	Logistics performance index: efficiency of customs clearance process (1 = low to 5 = high)	WDI
literacy_adult	Literacy rate, adult total (% of people ages 15 and above)	WDI
labforce_pr	Labor force participation rate, total (% of total population ages 15–64)	WDI
transport_invest	Investment in transport with private participation (current US$)	WDI
inflation	Inflation, consumer prices (annual %)	WDI
informalemp_Tot	Informal employment (total)	WDI
industry_VA	Industry (including construction), value added (% of GDP)	WDI
hci	Human capital index (HCI) (scale 0–1)	WDI
house_spend	Households and NPISHs final consumption expenditure (% of GDP)	WDI
grossavings	Adjusted annual gross savings (% of GNI)	WDI
natl_expend	National expenditure (% of GNI)	WDI
gfcf	Gross fixed capital formation (annual % growth)	WDI
domesticinvest	Gross fixed capital formation, private sector (% of GDP)	WDI
gov_educ	Expenditure on secondary education (% of government expenditure on education)	WDI
gov	General government final consumption expenditure (% of GDP)	WDI
gov_gdp	Government recurrent expenditure (%GDP)	WDI
gpc_grof	GDP per capita growth (annual %)	WDI
gpc	GDP per capita, PPP (constant 2017 international $)	WDI
gdpg	GDP growth (annual %)	WDI
fdi	Foreign direct investment, net inflows (% of GDP)	WDI
emp_ind	Employment in Employment in industry (% of total employment)	WDI
emp_agric	Employment in agriculture (% of total employment)	WDI
ease	Ease of doing business index (1 = most business-friendly regulations)	WDI
health_exp	Government health expenditure (%GDP)	WDI
cps	Private credit by deposit money banks and other financial institutions to GDP (%)	GFDD
cpia_transparency	CPIA transparency, accountability, and corruption in the public sector rating (1 = low to 6 = high)	CPIA
cpia_trade	CPIA trade rating (1 = low to 6 = high)	CPIA
cpia_socprotect	CPIA social protection rating (1 = low to 6 = high)	CPIA
cpia_publicmgt	CPIA public sector management and institutions cluster average (1 = low to 6 = high)	CPIA
cpia_socinclusion	CPIA policies for social inclusion/equity cluster average (1 = low to 6 = high)	CPIA
cpia_macro	CPIA macroeconomic management rating (1 = low to 6 = high)	CPIA
cpia_gender	CPIA gender equality rating (1 = low to 6 = high)	CPIA
cpia_finsector	CPIA financial sector rating (1 = low to 6 = high)	CPIA
debt	Public debt stock (%GDP)	WDI
moneyg	Broad money growth (annual %)	GFDD
agric_VA	Agriculture, forestry, and fishing, value added (% of GDP)	WDI
electricaccess_pop	Access to electricity (% of population)	WDI
electricaccess_rur	Access to electricity, rural (% of rural population)	WDI
importburden	Cost to import, documentary compliance (US$)	WDI
exportburden	Cost to export, documentary compliance (US$)	WDI
natresourcerent	Natural resource rent %GDP)	WDI
kofgidj	KOF. overall globalization index (de jure)	KOF. Index
kofecgj	KOF. economic globalization index (de jure)	KOF. Index
koftrgj	KOF. trade globalization index (de jure)	KOF. Index
koffindj	KOF. financial globalization index (de jure)	KOF. Index
kofsodj	KOF. social globalization index (de jure)	KOF. Index
gini	Gini index inequality indicators	GCIP
fin_devt	Financial development index	Findex
fi	Financial institutions index	Findex
fm	Financial markets index	Findex
fid	Financial institutions depth index	Findex
fia	Financial institutions access index	Findex
fie	Financial institutions efficiency index	Findex
fmd	Financial markets depth index	Findex
fma	Financial markets access index	Findex
fme	Financial markets efficiency index	Findex
npl	Bank nonperforming loans to gross loans (%)	GFDD
bankOHcost	Bank overhead costs to total assets (%)	GFDD
roa_net	Bank return on assets (%, after tax)	GFDD
roe_net	Bank return on equity (%, after tax)	GFDD
bankCrisis	Banking crisis dummy (1 = banking crisis, 0 = none)	GFDD
boone	Boone indicator. A measure of degree of competition based on profit-efficiency in the banking market	GFDD
onlinepayment	Electronic payments used to make payments (% age 15 +)	GFDD
insurancePrem	Life insurance premium volume to GDP (%)	GFDD
phonePayment	Mobile phone for paying bills online	GFDD
phoneMomo	Mobile phone penetration (able to perform mobile money transaction)	GFDD
remit	Remittance inflows (%GDP)	GFDD
stockPxVol	Stock price volatility index	GFDD
infrastr_qua	Infrastructure quality score	WDI
sse_gp	School enrolment, secondary (gross), gender parity index (GPI)	WDI
sis_m	Secure Internet servers (per 1 million people)	WDI
int_pop	Individuals using the Internet (% of population)	WDI
mcs_hd	Mobile cellular subscriptions (per 100 people)	WDI
fts_hd	Fixed telephone subscriptions (per 100 people)	WDI
fbs_hd	Fixed broadband subscriptions (per 100 people)	WDI
fd2	Square of financial development index	Generated
ps	Severity of poverty	Generated

*FD* index is financial development (International Monetary Fund), *GFDD* is global financial development database (Word Bank), *KOF* index is the Konjunkturforschungsstelle (KOF) index, *GCIP* is Global Consumption and Income Project, *CPIA* is Country Policy and Institutional Assessment (World Bank), and *WDI* is World Development Indicators (World Bank)

Source: authors’ construct, 2021

### Estimation Strategy

The empirical focus of this paper is in two parts. The first part is dedicated to the specification of the variable selection techniques while the inferential models are presented in the second part. In line with the objectives of the study, we do not employ traditional panel data estimation techniques for the analysis. For instance, the panel least squares estimator is inappropriate as it cannot explicitly perform variable selection from the 113 potential drivers of growth. Second, traditional methods such as the panel corrected standard errors and generalized method of moments cannot be relied upon as the presence of more predictors can cause the required matrix (${X}^{^{\prime}}X$) to be invertible. Even if it is possible, the presence of too many covariates may cause overfitting. In the presence of overfitting, although the attendant estimates are not biased, they are less efficient^8^ (James et al., 2013). This is due to the fact that as the covariates become large, least squares assumptions of no multicollinearity, homoscedasticity, and exogeneity typically break down, therefore overfitting the model. This causes the out of sample error to increase, making inference and predictions flawed (James et al., 2013).

Addressing this econometric concern can be through the application of machine learning regularization techniques, which are effective for variable selection regardless of the number of covariates, model specification, nonlinearity, and time (Tibshirani, 1996). In this study, therefore, we train recent machine learning regularization algorithms to learn patterns in the underlying dataset to identify the main drivers of economic growth. Regularization is done by utilizing the bias-variance trade-off, where a tuning parameter (i.e., the bias) is introduced to reduce the variance associated with large datasets and consequently yield sparse estimates. In specifics, we train algorithms for four alternative shrinkage models—the first three from the lasso family (i.e., the Standard lasso, the minimum Schwarz Bayesian information criterion lasso, and the Adaptive lasso) and the Elasticnet to achieve the first objective.^9^ Next, we perform causal inference on the selected covariates in objective 1 by running the lasso inferential models of double-selection linear lasso, partialing-out lasso linear regression, and partialing-out lasso instrumental variable regression to address objective 2. To this end, the STATA (version 16) and R (version 3.6) software are employed. The latter is employed primarily for data engineering and descriptive purposes while the data partitioning, regularization, and inferential estimates are carried out using the former.

#### Specification of Regularization Models

#### Specification of Standard Lasso and Minimum BIC Lasso Models

To address the ineffectiveness of traditional regression techniques in variable selection, Tibshirani (1996) introduced the standard lasso. Like other shrinkage techniques, the main advantages of the Standard lasso are that it (1) enhances the model interpretability by eliminating irrelevant variables that are not associated with the response variable; (2) enhances prediction accuracy, because shrinking and removing irrelevant predictors can reduce variance without a substantial increase in the bias; and (3) is limitless to data dimensionality.

In line with objective 1 of this study, the *Standard lasso* is applied to select the key drivers of economic growth by penalizing the model coefficients through a tuning parameter (λ) (Tibshirani, 1996; Belloni & Chernozhukov, 2014). Following Tibshiran (1996), we specify the objective function for the Standard lasso as shown in Eq. (1). For the Standard lasso algorithms to detect the key predicators of economic growth from a pool of several possible predictors, the penalty ($\lambda \sum_{j=1}^{\rho }|{\beta }_{j}|$), also referred to ${\mathcal{l}}_{1}$-norm, is introduced to obtain ${\widehat{\beta }}_{lasso}$ defined in Eq. (2):

1 $${Q}_{L}=\frac{1}{N}\sum\textstyle_{i=1}^{N}{\omega }_{i}f\left({y}_{it}, {\beta }_{0}+{X}_{it}{\beta }^{^{\prime}}\right)+\lambda \sum\textstyle_{j=1}^{p}{k}_{j}|{\beta }_{j}|$$

2 $${\widehat{\beta }}_{lasso}=min\left\{SSE+\lambda \sum\textstyle_{j=1}^{\rho }|{\beta }_{j}|\right\}$$

where ${y}_{it}$ is economic growth in country ***i*** in year ***t*** and ${X}_{it}$ is a vector of all the possible predictors of economic growth. The objective, therefore, is the minimization of the model sum of square errors with a given ${\mathcal{l}}_{1}$-norm. It is imperative to point out that if the tuning parameter, $\uplambda =0$, then we have a full model as in the least square estimator, while $\uplambda \to \infty$ is an intercept-only model. For brevity, we indicate that the specification of the *minimum BIC* lasso follows that of the *Standard lasso* with the same penalty and objective function but variable selection is based on the model with the least BIC (Schwarz, 1978). Some known drawbacks of these techniques are that, they (1) may become inconsistent as features grow rapidly and (2) are unable to perform hypothesis tests.

#### Specification of Adaptive Lasso Model

To enhance the consistency of regularization, Zou (2006) introduced the adaptive lasso technique, which in addition to the ${\mathcal{l}}_{1}$-norm penalty, adds the *oracle property* (${z}_{j}$). Relative to the Standard lasso, the *oracle property* enhances shrinkage or subset selection even when data attributes grow faster than the number of observations. In this study, we employ the Adaptive lasso technique as an alternative to the Standard lasso and minimum BIC lasso in addressing objective 1. Following Zou (2006), we minimize the objective function in (3) by applying the Adaptive lasso estimator (${\widehat{\beta }}_{AdaptiveLasso}$) specified in Eq. (4):

3 $${Q}_{L}=\frac{1}{N}\sum\textstyle_{i=1}^{N}{\omega }_{i}f\left({y}_{it}, {\beta }_{0}+{X}_{it}{\beta }^{^{\prime}}\right)+\lambda \sum\textstyle_{j=1}^{p}{k}_{j}|{\beta }_{j}|$$

4 $${\widehat{\beta }}_{AdaptiveLasso}=min\left\{SSE+\lambda \sum\textstyle_{j=1}^{\rho }{z}_{j}|{\beta }_{j}|\right\}$$

where ${y}_{it}$ is the outcome variable (economic growth) in country ***i*** in year ***t***, ${X}_{it}$ is a vector of all 113 covariates of economic growth, and ${\beta }^{^{\prime}}$ are the attendant parameters.

#### Specification of Elasticnet Model

The Elasticnet method draws on the strengths of the Standard lasso and ridge regression by applying the ${\mathcal{l}}_{1}$ and ${\mathcal{l}}_{2}$ penalization norms. The strength of the Elasticnet is that in highly correlated covariates, it can produce sparse and consistent regularization than the lasso family algorithms (Zou & Hastie, 2005). Also, with the application of the ${\mathcal{l}}_{1}$ and ${\mathcal{l}}_{2}$ penalization norms, the Elasticnet becomes flexible in subset selection. To perform variable selection, the Elasticnet estimator minimizes the objective function:

5 $${Q}_{en}=\frac{1}{N}\sum\textstyle_{i=1}^{N}{\omega }_{i}f\left({y}_{it}, {\beta }_{0}+{X}_{i}{\beta }^{^{\prime}}\right)+\lambda \sum_{j=1}^{p}{k}_{j}\left\{\frac{1-\alpha }{2}{\beta }_{j}^{2}+|{\beta }_{j}|\right\}$$

where ${y}_{it}$, ${X}_{i}$, and ${\beta }^{^{\prime}}$ are as defined in previous sections and *α* is an additional Elasticnet penalty parameter,^10^ which takes on values only in [0,1]. That is, sparsity occurs when 0 < α < 1 and λ > 0. This implies that in special cases, the Elasticnet plunges into either the ridge estimator (i.e., when λ = 0) or the Standard lasso estimator (i.e., when λ = 1).

#### Choice of Tuning Parameter

A fundamental concern regarding variable selection is the choice of the tunning parameter (**λ**). A good value of **λ** is essential for the overall performance of regularization models as it controls the strength of shrinkage and the concomitant prediction and inference (Schneider & Wagner, 2012). Among the widely used methods for choosing an efficient **λ** are cross validation (CV), Bayesian information criterion (BIC), and Akaike information criterion (AIC) (Tibshirani & Taylor, 2012). But it needs to be pointed out that if regularization becomes too strong, relevant variables may be omitted and coefficients may be shrunk excessively. Therefore, information criteria such as the BIC and AIC might be preferable to CV, since they are faster to compute and are less volatile in small samples (Zou et al., 2007). However, to the extent that setting **λ** under a researcher’s discretion can yield “target sparsity” and harm both predictive capacity and inferences (Hastie et al., 2019), we rely on BIC and CV^11^in determining **λ**.

#### Specification of Lasso Inferential Models

Since the aforementioned variable selection techniques do not provide estimates and confidence intervals essential for inference,^12^ we apply the lasso inferential techniques to provide robust estimates on the selected predictors of economic growth. In specifics, we run the double-selection lasso linear model (DSL), the partialing-out lasso linear regression (POLR), and the partialing-out lasso instrumental variable regression (POIVLR) using the selected covariates in objective 1 as the variables of interest and the unselected (redundant) variables as controls.

It is imperative to note that due to the sparsity of the regularization techniques, the control variables are usually many. In view of this, the lasso inferential models consider these controls as irrelevant, and therefore, their inferential statistics are not reported. However, the number of relevant controls and instruments are indicated as part of the general regression statistics. Further, unlike the variables of interest, which the researcher has no flexibility of adding or excluding from model, the researcher can indicate the number of controls in the model.^13^ The strength of these models is that they are built to produce unbiased and efficient estimates irrespective of data dimensionality, model specification, and multicollinearity.

#### Double-Selection Lasso Linear Model

In line with the second objective, we follow Belloni et al. (2014) by specifying the double-selection lasso (DSL) linear model as:

6 $$E\left[Y|d,x\right]=\psi {\alpha }^{^{\prime}}+{\phi \beta }^{^{\prime}},$$

where *y* is economic growth and is modeled to depend on $\psi$, containing *J* covariates of interest (i.e., the Elasticnet or lasso selected key drivers of economic growth) and $\phi$, which contains $p$ controls (i.e., the weak drivers of economic growth). The DSL estimator produces estimates on $J$ while relaxing the estimates for $p$.

#### Partialing-Out Lasso Linear Regression

In reference to the DSL, an added advantage of the partialing-out lasso linear regression (POLR) is that it enhances the efficacy of inference as the model becomes too complex. Following Belloni et al. (2012) and Chernozhukov et al. (2015), we specify the POLR estimator as:

7 $$E\left[Y|d,x\right]=d{\alpha }^{^{\prime}}+{X\beta }^{^{\prime}},$$

where $y$ is outcome variable (economic growth), $d$ is a vector containing the $J$ predictors of interest (i.e., the nonzero selected covariates of economic growth), and $X$ contains the $p$ controls (i.e., the unselected predictors of economic growth). Like the DSL, the POLR yields estimates, standard errors, and confidence intervals on the $J$ covariates while relaxing that of the $p$ controls.

#### Partialing-Out Lasso Instrumental Variable Regression

In large data regression problems like this study, sources of endogeneity abound largely due to bi-causality. For example, endogeneity can arise from the argument articulated in the supply-leading and demand-following hypotheses concerning financial development and economic growth (King & Levine, 1993). To address this, we follow Chernozhukov et al. (2015) by performing a partialing-out lasso instrumental variable regression (POIVLR). The POIVLR is specified as:

8 $$y=\Psi {\alpha }_{d}^{^{\prime}}+\Phi {\alpha }_{f}^{^{\prime}}+{X\beta }^{^{\prime}}+ \varepsilon ,$$

where $y$ is economic growth;$\Psi$ comprises ${J}_{d}$ endogenous covariates of interest; $f$ contains the ${J}_{f}$ exogenous covariates of interest; and $X$ contains ${\mathcal{p}}_{x}$ controls. Allowing for potential endogeneity primarily due to simultaneity, ${\mathcal{p}}_{z}$ outside instrumental variables denoted by $z$ that are correlated with $d$ but not with $\varepsilon$ are introduced. As aforesaid, the simultaneity between financial development and economic growth presents endogeneity concerns which are addressed using the $z$ instruments.^14^ Theoretically, the controls and instrument can grow with the sample size; however, $\beta$ and nonzero coefficients in $z$ must be sparse.

### Data Engineering and Partitioning

One of the key requirements of effective regularization is that the underlying dataset is strongly balanced. To this end, we employ the *K-nearest neighbor* (KNN) data imputation technique to address missing observations, particularly for variables such as the policy and institutional indicators (see Fig. 10). The KNN follows the principle that developments regarding variables drawn from a similar population exhibit similar properties (Van Hulse & Khoshgoftaar, 2014). In principle, the KNN selects the nearby neighbors based on a distance metric and estimates the missing observation with the attendant mean or mode. It is worth noting that while the mean rule is used to address missing observations in numerical variables, the latter is employed to address missing observations in categorical variables (Pan et al., 2015). Per this principle, this study relies on the mean rule, which uses the Minkowski distance as specified in Eq. (9) in addressing the missing observations.

9 $$d\left(i,j\right)={({\left|{x}_{i1}-{x}_{j1}\right|}^{q}+{\left|{x}_{i2}-{x}_{j2}\right|}^{q}+\dots +{\left|{x}_{i\rho }-{x}_{j\rho }\right|)}^{q}}^{1/q},$$

where $q$ is the Minkowski coefficient, $d\left(i,j\right)$ is the Minkowski distance for observations *i* and *j*, and $x$ are the variables. That said, we follow Ofori et al. (2022) by partitioning the dataset into two parts—the training set (70%) and testing test (30%) samples. We do this by applying the simple random and stratified data splitting techniques. In line with Ofori et al. (2022), we take cues from James et al. (2013) that among all other possible sets, the 70–30 and 80–20 splits are the data partitioning sets allowing reasonable representation of all variables in both the training and testing samples.

## Presentation and Discussion of Results

### Exploratory Data Analysis

For brevity, the exploratory data analysis is limited to the data partitioning results,^15^ the distribution of economic growth, and the summary statistics. Information gleaned from the summary statistics in Table 2,^16^ shows an average economic growth (i.e., real GDP growth rate) value of 3.58 percent in the training set as compared to 3.95 percent in the testing set. Also, the average trade openness value as a percentage of GDP is 67.48 in the training set compared to 66.85 percent in the testing set. Additionally, we observe a mean unemployment rate of 7.58 percent in the training set compared to 7.68 percent in the testing set. It is also evident from Table 2 that the average transparency, accountability, and corruption score of 2.81 and 2.8 in the training and testing sets, respectively. Finally, Fig. 9 in the Appendix shows that 99.9 percent observations were present in the dataset before the data imputation (see the data engineering results in Fig. 10 in the Appendix).

**Table 2:** Summary statistics for training and testing sets

Variable	Obs	Mean (training set)	Std. Dev. (training set)	Min (training set)	Max (training set)	Mean (testing set)	Std. Dev. (Testing set)	Min(Testing set)	Max (Testing set)
wagessalary	1204(516)	26.779	21.77	5.049	85.412	27.151	22.537	5.106	85.871
vul tot	1204(516)	71.106	22.471	9.429	94.75	70.747	23.268	8.826	94.759
lossesdue power	1204(516)	6.804	4.938	0.7	25.1	6.427	4.718	0	25.1
urban pop	1204(516)	4.268	1.885	-6.879	17.499	4.195	1.888	-7.182	15.714
unempl	1204(516)	7.58	7.48	0.3	37.976	7.684	7.513	0.3	37.94
trade	1204(516)	67.487	36.253	9.136	290.499	66.853	34.662	0	311.354
trade tax	1204(516)	16.15	13.095	0.107	63.451	15.404	12.787	0	63.451
taxrev	1204(516)	14.198	6.339	4.204	39.258	14.182	6.287	0	37.353
tariffwm	1204(516)	12.301	5.381	0.84	32.6	12.771	7.106	0	91.27
self_employ	1204(516)	73.221	21.77	14.588	94.951	72.849	22.537	14.129	94.894
rurpopgrof	1204(516)	1.735	1.335	-6.707	10.906	1.656	1.477	-7.866	7.297
rd	1204(516)	0.275	0.18	0.005	0.888	0.28	0.18	0	0.898
rer	1204(516)	198.423	168.865	46.021	3520.534	200.184	122.564	0	2182.799
hiv_preva	1204(516)	2.664	3.554	0.1	24.1	2.584	3.74	0	24.2
prenatal	1204(516)	77.749	17.935	23.4	99.4	75.969	19.565	0	99.3
poverthc	1204(516)	48.929	13.849	7.9	73.2	47.661	14.206	7.9	73.2
povertyhc mid	1204(516)	69.272	23.899	2.2	98.5	68.718	24.645	3.1	98.5
povertyhc low	1204(516)	49.48	24.837	0.2	94.3	49.342	25.332	0.4	94.3
povmidd	1204(516)	38.282	18.955	0.4	86.7	38.317	19.581	0.7	86.7
povint	1204(516)	23.018	16.497	0	86.7	23.342	17.313	0.1	86.7
urbanization	1204(516)	39.323	14.338	10.838	92.697	38.745	13.696	0	100
popgrof	1204(516)	2.614	0.875	-1.305	7.449	2.532	1.101	-6.766	8.118
finaninsti	1204(516)	2.623e + 08	9.764e + 08	-2.027e + 09	8.594e + 09	2.503e + 08	9.760e + 08	-7.162e + 09	6.988e + 09
exr	1204(516)	408.351	1250.663	0	19,068.417	407.802	1462.092	0	18,498.601
noda	1204(516)	11.632	11.713	-0.188	79.827	11.091	11.397	-0.251	94.946
netmigration	1204(516)	-21,467.614	262,740	-1,374,270	1,457,943	-19,246.359	295,523.94	-1,374,270	1,457,943
mortality 5yrs	1204(516)	127.098	63.887	13.7	329.3	127.848	68.421	0	337.4
manuf VA	1204(516)	2.456	9.168	-34.921	97.709	3.636	16.181	-43.84	375.158
logisticqua TT	1204(516)	2.167	0.335	1.27	3.79	2.132	0.349	0	3.79
logisticquaoveral	1204(516)	2.398	0.302	1.61	3.775	2.374	0.321	0	3.67
logisticqua ship	1204(516)	2.845	0.431	1.67	4.03	2.839	0.458	0	4.03
literacy adult	1204(516)	56.851	21.081	10.895	95.868	56.709	21.682	0	95.868
labforce pr	1204(516)	69.802	11.312	42.381	92.453	69.87	11.536	42.409	92.453
transport invest	1204(516)	3.050e + 08	5.834e + 08	0	3.483e + 09	3.543e + 08	6.454e + 08	0	3.483e + 09
inflation	1204(516)	45.776	822.219	-9.809	23,773.132	17.538	102.741	-13.057	2154.437
industry VA	1204(516)	22.678	12.112	1.305	72.153	23.735	12.355	0	72.717
hci	1204(516)	0.393	0.069	0	0.678	0.396	0.077	0	0.678
house spend	1204(516)	0.713	8.947	-45.41	65.181	1.032	8.214	-46.068	87.014
grossavings	1204(516)	15.406	18.256	-69.534	84.49	17.083	16.674	-70.263	87.096
natl expend	1204(516)	109.86	18.643	57.699	255.256	108.993	16.652	0	261.428
gfcf	1204(516)	21.085	10.653	-2.424	85.941	21.436	10.946	0	93.547
domesticinvest	1204(516)	11.914	20.269	-133.979	85.541	13.388	18.583	-141.974	88.389
gov_educ	1204(516)	15.581	5.659	4.673	37.521	15.445	5.567	0	34.309
gov	1204(516)	6.38	31.534	-71.464	565.539	4.477	18.399	-68.238	165.168
gov_gdp	1204(516)	14.813	6.874	0	51.975	14.894	6.773	0	51.975
gpc	1204(516)	3783.244	4347.165	464.018	29,223.465	3855.974	4458.085	0	27,242.656
gdpg	1204(516)	3.588	4.881	-30.145	33.629	3.592	5.522	-50.248	35.224
fdi	1204(516)	3.353	7.973	-8.703	103.337	2.435	4.215	-28.624	40.167
emp ind	1204(516)	12.601	8.19	1.43	42.939	12.711	8.62	1.539	43.114
emp agric	1204(516)	54.751	21.465	4.6	92.298	54.951	22.264	4.65	92.303
electricity	1204(516)	545.412	953.395	0	4665.176	533.118	958.839	0	4851.693
ease	1204(516)	136.628	38.692	13	184	134.261	41.851	0	184
health exp	1204(516)	1.661	1.098	0.062	5.496	1.631	1.102	0	6.049
cps	1204(516)	17.836	20.968	0.403	149.234	18.695	21.393	0	160.125
cpia transparency	1204(516)	2.812	0.592	1.5	4.5	2.808	0.6	0	4.5
cpia trade	1204(516)	3.728	0.522	2	4.5	3.743	0.511	0	4.5
cpiapublicmgt	1204(516)	3.006	0.455	2	4.1	3.02	0.478	0	4
cpiasocinclusion	1204(516)	3.157	0.471	2.2	4.3	3.166	0.477	0	4.3
cpia macro	1204(516)	3.641	0.66	1.5	5	3.671	0.645	0	5
cpia gender	1204(516)	3.195	0.535	2	4.5	3.196	0.555	0	4.5
cpiafinsector	1204(516)	2.954	0.428	2	4	2.947	0.437	0	4
debt	1204(516)	104.141	103.919	0	289.845	109.07	105.418	0	289.845
moneyg	1204(516)	66.543	455.289	-99.864	6968.922	76.271	457.558	-29.245	4105.573
agric VA	1204(516)	26.747	16.109	1.828	76.534	25.563	15.59	0	79.042
importburden	1204(516)	137.893	100.549	3.5	420	136.994	107.926	0	588
exportburden	1204(516)	108.208	71.445	4	347	107.994	77.735	0	515
natresourcerent	1204(516)	10.836	9.99	0	56.939	10.861	10.305	0	59.604
kofgi	1204(516)	40.353	10.119	16.922	72.354	39.856	10.381	0	72.269
kofgidj	1204(516)	40.995	11.306	13.308	80.993	40.575	11.51	0	81.288
kofecgj	1204(516)	34.676	10.965	10.514	81.49	34.073	11.136	0	79.549
koftrgj	1204(516)	28.778	14.291	6.494	88.014	28.567	14.566	0	88.497
koffindj	1204(516)	40.481	14.338	6.099	80.37	39.63	13.921	0	81.357
kofsodj	1204(516)	35.241	16.046	4.289	84.779	34.64	16.833	0	85.141
gini	1204(516)	51.994	21.443	0	86.276	51.909	21.048	0	86.832
fin_devt	1204(516)	0.124	0.089	0	0.641	0.124	0.088	0	0.648
fi	1204(516)	0.209	0.125	0	0.74	0.211	0.126	0	0.73
fm	1204(516)	0.034	0.072	0	0.52	0.031	0.068	0	0.54
fid	1204(516)	0.099	0.149	0	0.88	0.095	0.144	0	0.88
fia	1204(516)	0.075	0.125	0	0.88	0.078	0.132	0	0.86
fie	1204(516)	0.49	0.197	0	0.99	0.499	0.208	0	0.98
fmd	1204(516)	0.052	0.1	0	0.83	0.045	0.09	0	0.75
fma	1204(516)	0.031	0.11	0	0.89	0.032	0.112	0	0.58
fme	1204(516)	0.016	0.058	0	0.96	0.014	0.046	0	0.42
npl	1204(516)	13.187	12.73	0	74.1	13.844	13.578	0	74.1
bankOHcost	1204(516)	6.471	5.082	0	89.423	6.159	4.073	0	28.192
roe net	1204(516)	17.975	24.454	-93.62	146.913	19.74	24.961	-93.62	160.344
roa net	1204(516)	1.675	2.837	-15.047	9.182	1.814	2.8	-15.047	12.106
Boone	1204(516)	-0.048	0.233	-2.541	1.607	-0.031	0.235	-0.896	1.607
onlinepayment	1204(516)	21.345	18.123	0	76.411	20.576	17.807	0	76.411
insurancePrem	1204(516)	0.633	1.79	0	14.52	0.622	1.72	0	15.381
phonePayment	1204(516)	3.801	5.157	0	37.105	3.626	5.141	0	37.105
phoneMomo	1204(516)	10.532	13.179	0	50.122	10.009	12.84	0	50.122
remit	1204(516)	4.666	19.582	0	235.924	4.057	15.585	0	232.217
stockPxVol	1204(516)	11.08	5.729	0	43.1	10.949	5.513	0	34.376
infrastr qua	1204(516)	3.467	0.749	1.8	5.417	3.432	0.784	0	5.641
ssegp	1204(516)	0.737	0.281	0	1.527	0.728	0.285	0	1.504
int pop	1204(516)	4.942	10.76	0	62	4.937	10.667	0	64
sis m	1204(516)	495.477	9655.395	0	264,256.63	302.971	5725.258	0	155,191.3
mcshd	1204(516)	23.577	38.438	0	198.152	23.21	38.546	0	173.811
fts hd	1204(516)	2.019	4.51	0	32.669	2.256	5.074	0	34.273
fbshd	1204(516)	0.299	1.767	0	27.603	0.322	1.717	0	21.639
fd2	1204(516)	0.023	0.044	0	0.41	0.023	0.042	0	0.42
ps	1204(516)	16.016	21.48	0	169.299	17.132	23.363	0.001	169.299

1204 is number of observations in training set; 516 is observations in testing set

Source: authors’ construct 2021

#### Data Partitioning and Distribution of Economic Growth

**Fig. 2:**
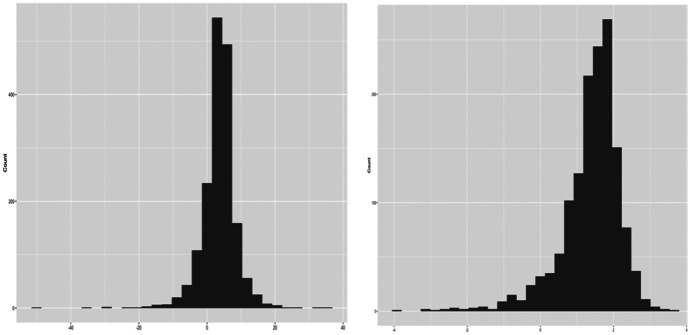
Distribution of economic growth at level (left) and its log-transformation (right)

A major decision regarding regularization is the form the outcome variable takes—either level or log transformed. On the latter, the distribution of economic growth as we show in Fig. 2 (right) is right-skewed. However, at level, as shown in Fig. 2 (left), economic growth is more symmetric and less heavy-tailed. At the backdrop that skewed distribution can have dire implications for regularization and the attendant inferential statistics, we run our shrinkage models using economic growth at level. Further, though non-standardization of covariates of economic growth does not constrain regularization, it is essential for ensuring the internal consistency of the data and comparability of the covariates. In view of this, the standardize option is invoked.

**Fig. 3:**
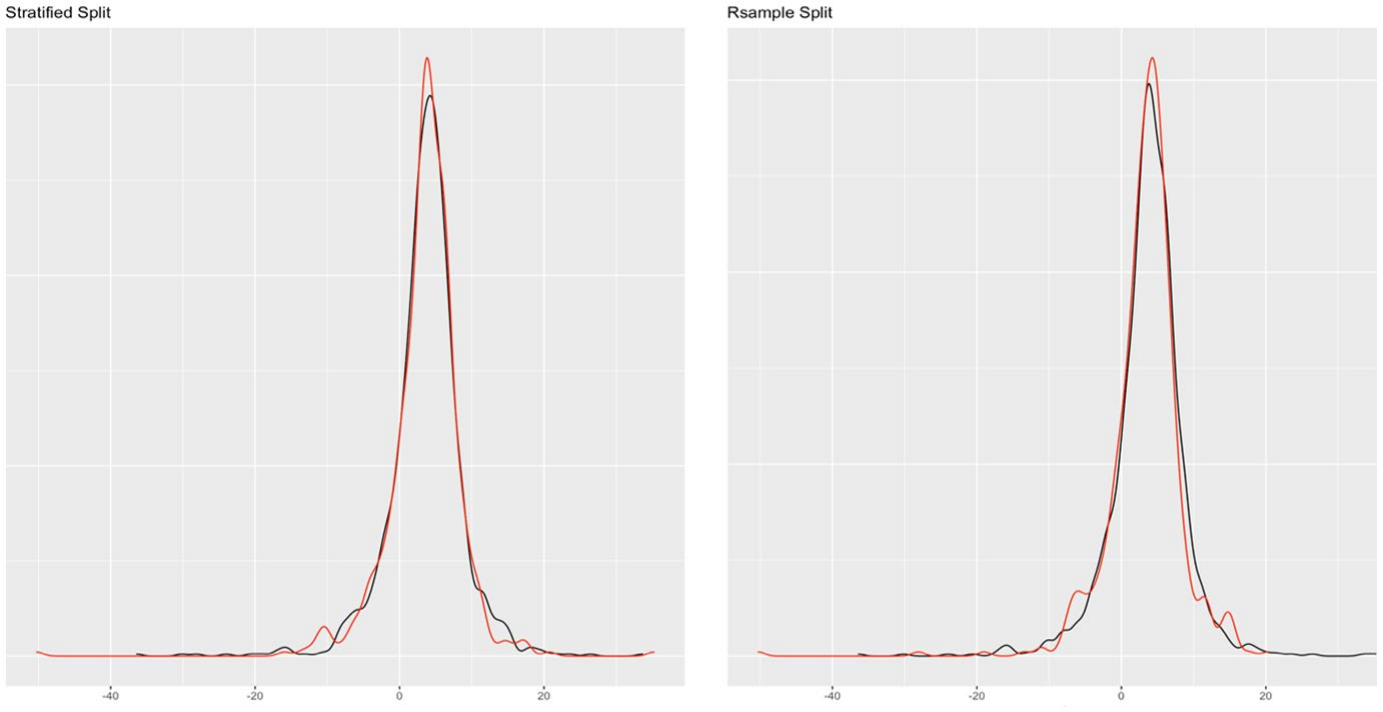
Data partitioning plot (base R method), training (black), and test (red)

On data partitioning, we perform a 70–30 split of the dataset using the *stratified method* (see Fig. 3 (left)). Additionally, in checking the reliability or consistency of the stratified split, we run the simple random data splitting technique, which yields similar results (Fig. 3 (right)).

### Regularization Results on the Main Drivers of Economic Growth in SSA

**Fig. 4:**
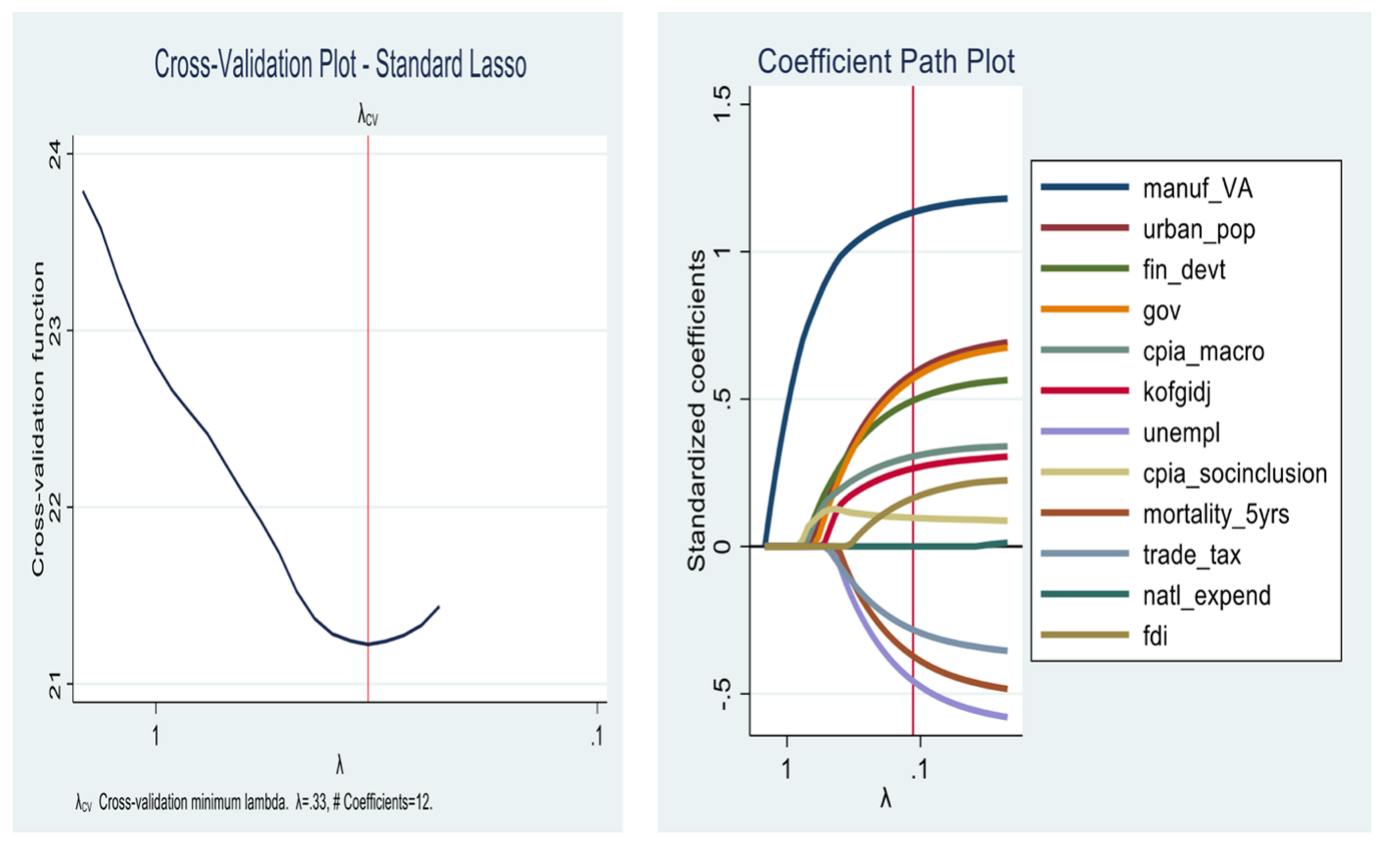
Cross-validation plot (left) and coefficient path plot (right) for standard lasso

**Fig. 5:**
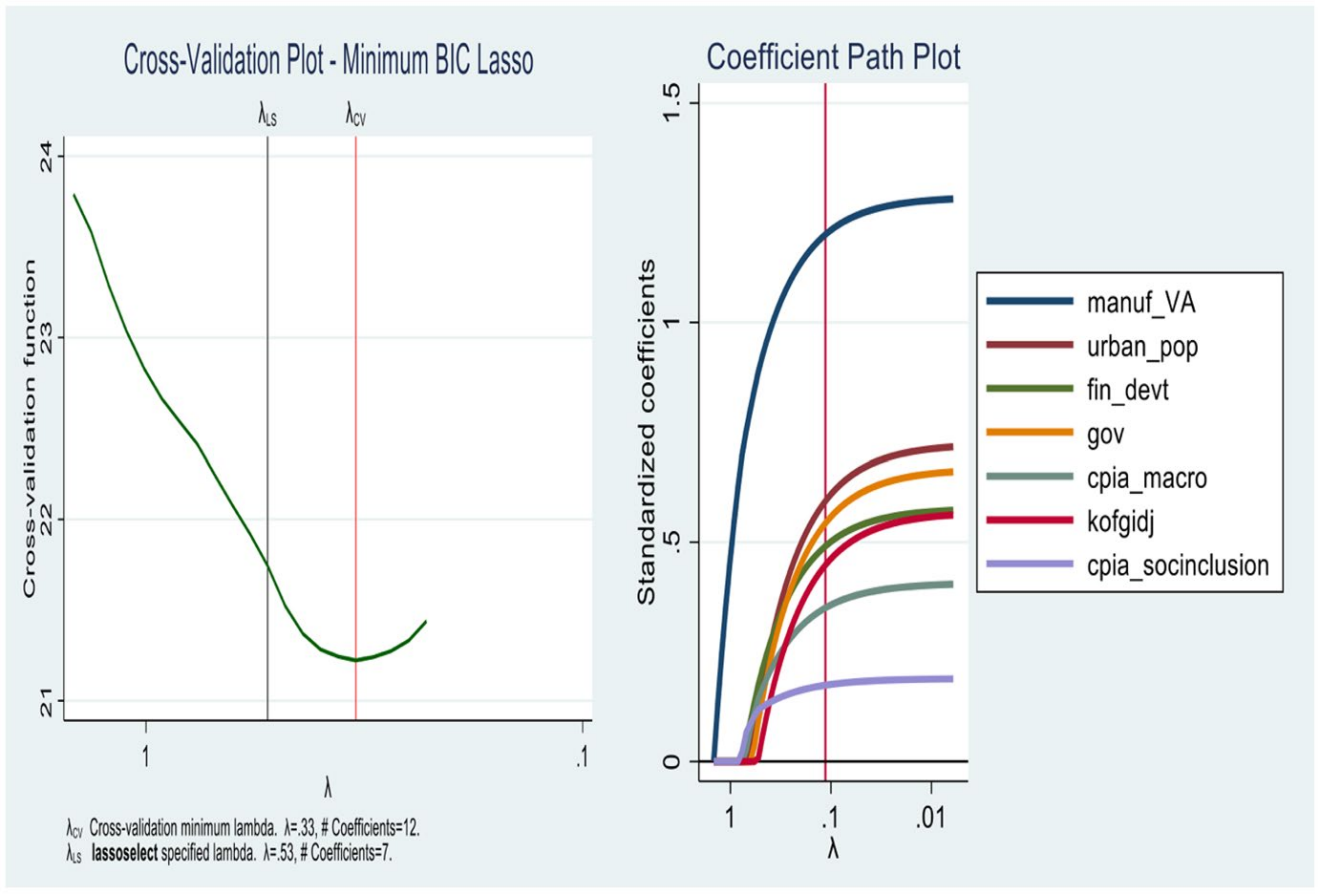
Cross-validation plot (left) and coefficient path plot (right) for minimum BIC lasso

**Fig. 6:**
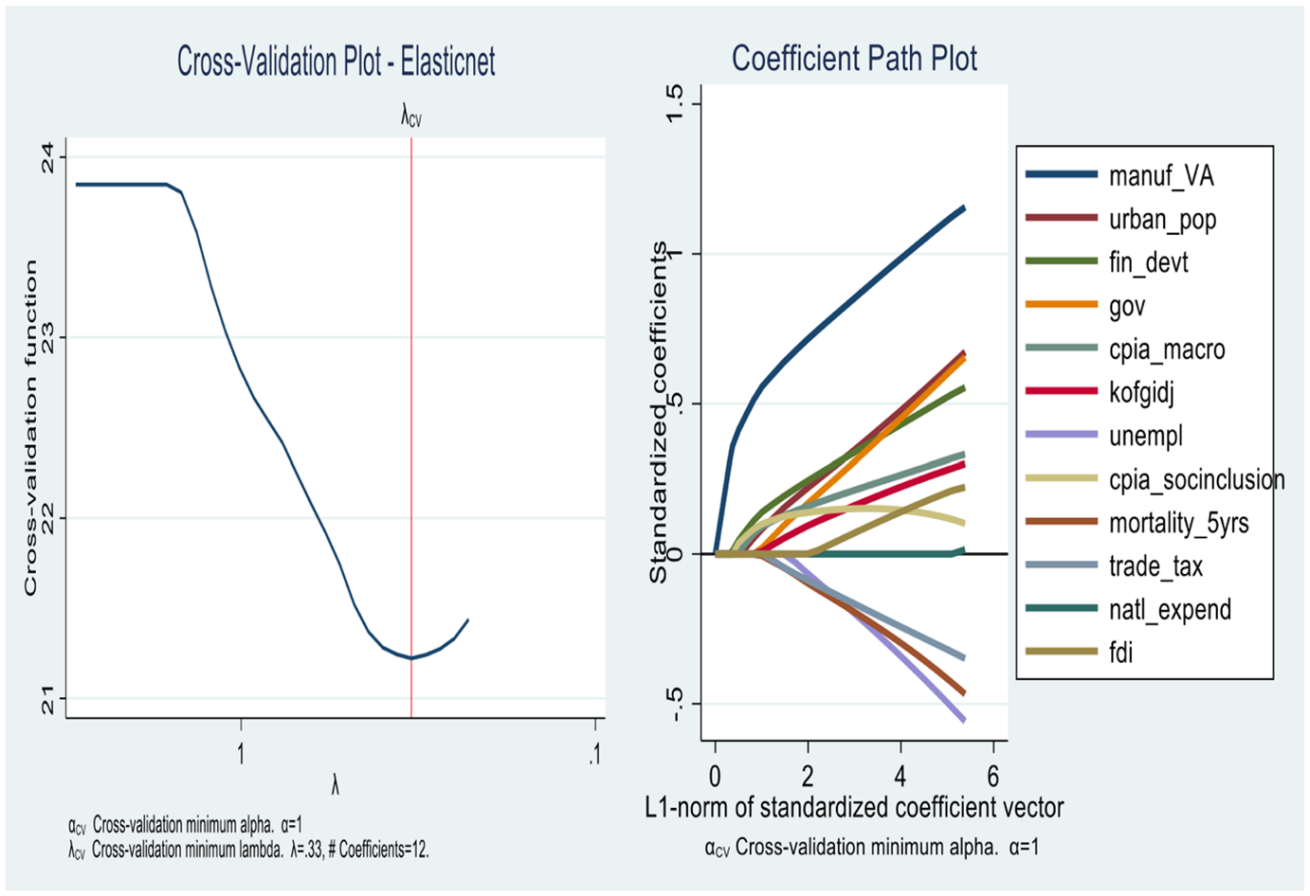
Cross-validation plot (left) and coefficient path plot (right) for ElasticNet

**Fig. 7:**
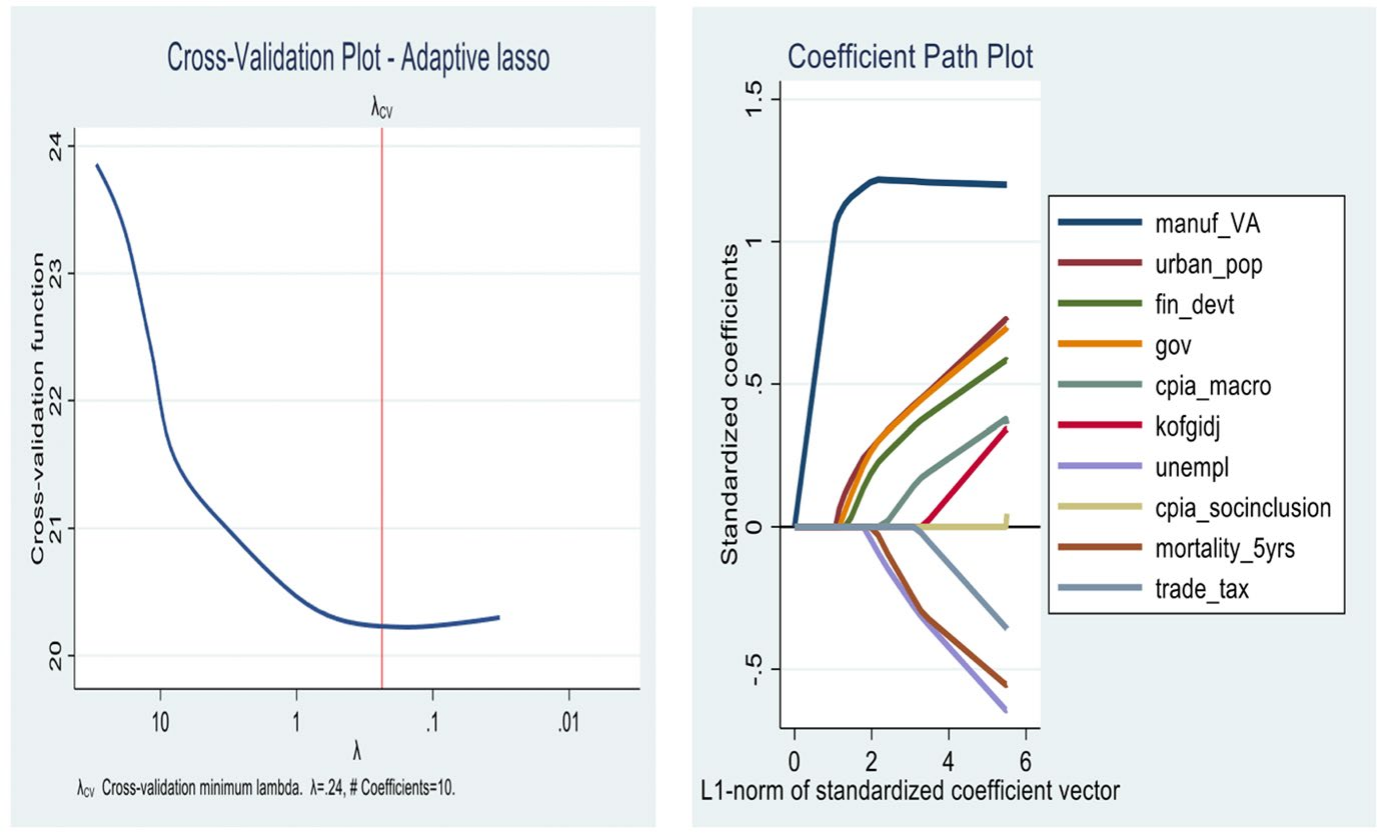
Cross-validation plot (left) and coefficient path plot (right) for adaptive lasso

**Fig. 8:**
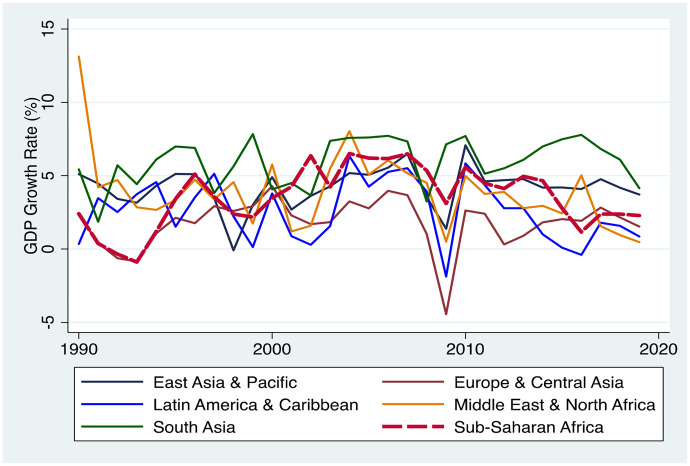
Trend of GDP Growth Across Regions, 1990 – 2019. Source: authors’ construct, 2021

**Fig. 9:**
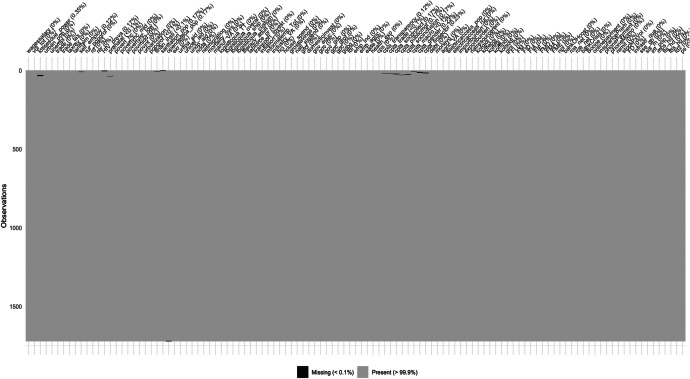
Overview of the dataset before data engineering. Source: authors’ construct (2021)

**Fig. 10:**
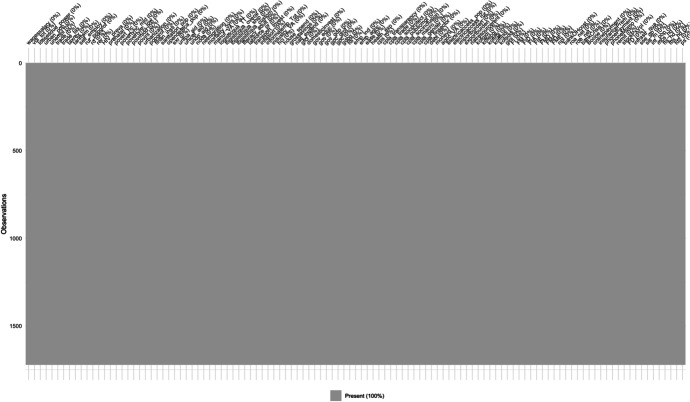
Overview of the dataset after data engineering. Source: Authors’ construct (2021)

In this section, the results for the first objective are presented. As we show from Figs. 4, 5, 6 and 7 the lassos and Elasticnet algorithms select different non-zero coefficients (i.e., predictors) of economic growth. We find that the *Standard lasso* selects 12 covariates as key drivers of economic growth under a ten-fold cross-validation tuning parameter ($\uplambda$) value of 0.33 (see Fig. 4). Further, the *Adaptive lasso* selects only 10 covariates from the total 113 as chief drivers of economic growth in SSA with a tuning parameter ($\uplambda$) value of 0.24. Similarly, we find a special case for the Elasticnet regularization as it selects covariates based on a minimum cross-validation lambda of 0.33 and a minimum cross-validation alpha of 1. While the Elasticnet plunges into the *Standard lasso* (i.e., selects 12 non-zero predictors), we find a sparser regularization in the *minimum BIC lasso* as it selects only 7 covariates of the total 113.

In Table 3, a detailed output of how covariates enter and leave the respective shrinkage models is presented. The results from the minimum BIC lasso, which yields the best regularization indicates that the key drivers of economic growth in SSA are *manufacturing (value addition)*, *population*, *financial development*, *government spending*, *macroeconomic management*, *globalization*, and *social inclusion*. The appropriateness of the results is evident in the post-estimation tests of cross-validation and coefficient path plots associated with each model (Figs. 4, 5, 6 and 7).

**Table 3:** Variable selection in regularization models

	Standard_lasso	Minimum_BIC_lasso		Elastic_Net	Adaptive_lasso
manuf_VA	x	x		x	x
urban_pop	x	x		x	x
gov	x	x		x	x
house_spend	x	x		x	x
cpia_macro	x	x		x	x
kofecdj	x	x		x	x
unempl	x		x		x
cpia_socinclusion	x	x		x	x
mortality_5yrs	x		x		x
trade_tax	x		x		x
natresourcerent	x		x		
fdi	x		x		
_cons	x	x		x	x

Legend: o Omitted, x estimated

Source: authors’ construct 2021

### Inferential Results for the Main Drivers of Economic Growth in SSA

In this section, the estimates on the 7 covariates of growth identified in objective 1 are provided. The results, which are reported in Table 4 are based on the DSL, POLR, and POIVLR estimation techniques, meaning that they are robust to heteroskedasticity, endogeneity, and model misspecification. To inform policy appropriately, we run three separate results for the (i) full sample, (ii) low-income countries, and (iii) middle- and high-income countries.

**Table 4:** Lasso estimates on the main drivers of economic growth in sub-Saharan Africa

	**All countries**			**Low income**	**Countries**		**Middle**	**High income**	**Countries**
	**(1)**	**(2)**	**(3)**	**(4)**	**(5)**	**(6)**	**(7)**	**(8)**	**(9)**
**Variables**	**DSL**	**POLR**	**POIVLR**	**DSL**	**POLR**	**POIVLR**	**DSL**	**POLR**	**POIVLR**
Manufacturing value addition	0.062*	0.066*	0.066*	0.125***	0.114***	0.114***	0.008	0.010	0.010
	(0.034)	(0.034)	(0.034)	(0.028)	(0.024)	(0.024)	(0.007)	(0.008)	(0.008)
Population (urban)	0.273***	0.309***	0.255**	0.318**	0.349**	0.349**	0.110	0.152	0.152
	(0.089)	(0.104)	(0.114)	(0.155)	(0.170)	(0.170)	(0.300)	(0.264)	(0.264)
Financial development	0.142***	0.133***	0.144***	0.165***	0.142***	0.142***	0.040	0.047	0.047
	(0.031)	(0.027)	(0.030)	(0.047)	(0.045)	(0.045)	(0.034)	(0.030)	(0.030)
Government expenditure	0.032***	0.034***	0.065**	0.058***	0.059***	0.059***	0.036*	0.023	0.023
	(0.011)	(0.011)	(0.029)	(0.020)	(0.018)	(0.018)	(0.020)	(0.022)	(0.022)
Macroeconomic management	0.757***	0.654**	0.737***	1.493***	0.825**	0.825**	0.885*	0.602	0.602
	(0.274)	(0.261)	(0.269)	(0.511)	(0.371)	(0.371)	(0.497)	(0.438)	(0.438)
Globalization	0.090**	0.078*	0.063	0.002	0.019	0.019	− 0.002	0.036***	0.036***
	(0.041)	(0.041)	(0.042)	(0.031)	(0.028)	(0.028)	(0.023)	(0.013)	(0.013)
Social inclusion	3.308***	2.497***	3.007***	− 1.502	− 1.017	− 1.017	3.231**	1.352	1.352
	(0.904)	(0.825)	(1.006)	(1.447)	(1.222)	(1.222)	(1.425)	(1.045)	(1.045)
Observations	1,720	1,720	1,720	798	798	798	350	350	350
Variables of interest	7	7	7	7	7	7	7	7	7
Controls	56	56	56	66	66	66	66	66	66
Controls selected	39	39	46	25	25	25	27	27	27
Instruments	–	–	12	–	–	7	–	–	7
Wald statistics	89.43***	81.72***	78.11***	38.22***	49.72***	49.72***	16.28**	24.29***	24.29***
Wald *p* value	0.000	0.000	0.000	0.000	0.000	0.000	0.022	0.001	0.001
Countries	42	42	42	20	20	20	22	22	22

*DSL* Double-selection lasso, *POLR* partialing-out linear lasso regression, *POIVLR* partialing-out instrumental variable linear regression. Robust standard errors in parentheses

*p < 0.1; **p < 0.05; ***p < 0.01;

To begin with, we find that manufacturing value addition matters for economic growth in SSA*.* The results show that a 1 percent increase in manufacturing value addition boosts economic growth by 0.06 percent. Across the low-income and middle-income divide, however, we find that manufacturing value addition is significant only in the case of the former. The evidence suggests that with appropriate economic governance, it is low-income countries that can make remarkable strides in economic growth through enhanced manufacturing value addition considering the implementation of the AfCFTA. This is more so as improvement in manufacturing can spur forward and backward linkages as well as global value chain participation.

Further, the results show that although financial development is directly related to economic growth in both low-income and middle-income countries, it is statistically significant only in the former. In terms of magnitudes, the results suggest that for every 1-point increase in financial development, economic growth rises by 0.14 percent in low-income countries. The effect of financial development is remarkable, suggesting that access to financial products and services can propel the huge informal sector of low-income countries to realize their innovative and entrepreneurial objectives. This is more so considering the fact that lags in financial access are glaring in the low-income countries compared to middle-income countries.

Additionally, we find that economic globalization drives economic growth in SSA. In the remit of low-income and middle-income countries, however, we find that economic integration matters for growth only in the case of the latter. The plausible explanation for this is that, relative to low-income countries, middle-income countries have made remarkable strides in developing their manufacturing base, coupled with a good absorptive capacity that can enable them to gain significantly from economic globalization. Albeit statistically insignificant, the positive relationship between growth and economic globalization for low-income countries provides sheer optimism considering the implementation of the AfCFTA and the expected rebound of FDI to Africa from 2022.

The result on economic globalization is linked to the remarkable finding on macroeconomic management. There is strong empirical evidence to show that every 1-point increase in the score of macroeconomic management boosts economic growth by 0.73 percent (column 3). This result is even strong (i.e., 0.82%) in the case of low-income countries (column 6). Indeed, one of the major problems of the region has been poor macroeconomic management often resulting in bailouts by foreign institutions.^17^ Although these bailouts have proved effective in propelling beneficiary countries toward prudent macroeconomic management paths, gains are mostly disrupted following exist, signifying the need for sustained commitment to fiscal and monetary discipline in SSA.

Also, we find that government expenditure is instrumental for economic growth in SSA. The result shows that a 1 percent increase in government expenditure boosts economic growth by 0.06 percent. However, this evidence is only significant in low-income SSA countries. A possible explanation for this is that, in middle-income countries, a high percentage of government expenditure goes into the recurrent expenditure compared to capital expenditure.

Moreover, we find that urban population matters for economic growth in the SSA. Additionally, the result reveal that urbanization is effective in fostering growth in the low-income countries compared to middle-income countries. This evidence appeals to logic in that economic activities driving growth in low-income SSA countries are mostly concentrated in urban centers. The result is in line with a World Bank (2009) report which argues that urban concentration is crucial in fostering growth in economies at the early stages of development. There is also the supporting evidence of gains from urbanization in that it reduces poverty and inequalities in opportunities, services, assets (Sekkat, 2017), and income inequality (see Oyvat, 2016).

Also, we provide strong empirical evidence to show that improving the coverage of social inclusion polices promotes economic growth in SSA by 3 percent (column 3). The result suggests that rolling out social intervention programs can propel SSA countries towards sustained growth trajectories. This is more so as social inclusion policies can build private sector capacity to withstand socioeconomic shocks. This is however not effective for growth in the low-income countries. This is also possible since institutions for developing human capital in these settings are weak, thereby providing little or no growth gains for such expenditure.

## Conclusion and Policy Recommendations

The study contributes to the economic growth literature on SSA by employing recent advances in machine learning to identify the key drivers of growth. In doing so, we train algorithms for four machine learning regularization models—the Standard lasso, the minimum BIC lasso, the Adaptive lasso, and the Elasticnet based on a dataset spanning 1980–2019 for 42 African countries. Our results show that machine learning techniques are powerful and effective in reducing model complexities associated with large-data regression problems. In this study, while both the Standard lasso and Elasticnet techniques select 12 covariates as the main determinants of economic growth, the minimum BIC lasso selects only 7 out of the total 113 possible predictors. The uniqueness of the study is that it presents policymakers interested in the SSA growth agenda, variables to target to foster and sustain growth. These variables are manufacturing (value addition), urban population, financial development, government spending, macroeconomic management, economic globalization, and social inclusion.

For middle-income SSA countries, we suggest the following recommendations. First, in line with the implementation of the AfCFTA and the green growth agenda of the SSA, it is recommended that policymakers invest strategically in the manufacturing sectors of their economies. This can prove crucial in turning around the slow growth trajectories of the region as economic globalization can spur the industrialization through forward and backward linkages, innovation diffusion, and global value chain participation. Policymakers are therefore advised to build the technical workforce of their economies to make sense of the knowledge and innovation transfers associated with economic integration. Second, to improve the ability, opportunities, and dignity of the marginalized to contribute meaningfully to national development, policymakers are to invest strategically in areas such as health, education, and vocational training. This is more so as ICT diffusion can reduce inequalities in accessing information and high cost of accessing opportunities due to polarization of administrative procedures in the SSA.

For low-income countries, efforts should be made to develop the financial sector. This could prove crucial for efficient resource allocation, which can be a gamechanger in spurring the industrialization agenda of the region thorough competition, innovation, dynamism, and enhanced global value chain participation. Resources should thus be channeled towards the development of payment system platforms and services, financial innovation, and information flow on consumers. In this regard, institutions interested in SSA’s development agenda such as the African Development Bank, the IMF, and the World Bank should provide technical and logistical support to aid the transformation of the region’s predominantly low productive informal sector to a more dynamic, highly competitive and export-oriented one.

Additionally, we recommend that policymakers commit to prudent macroeconomic management. We reckon that in a setting like SSA where vulnerabilities are widespread, sound macroeconomic management will prove momentous in mitigating the welfare setbacks imposed by socioeconomic shocks (e.g., Covid-19) while lessening the impact of future ones. This also calls for the need to channel resources into productive expenditure like infrastructure and energy supply, which could contribute to ensuring that economic globalization propel these countries sustained growth trajectories.

The study leaves room for future works. First, considering the contributions this study makes through machine learning techniques, the academic community can also draw on similar techniques, for instance, to identify factors key for analyzing poverty and inequality. Second, these techniques can be employed to examine whether the growth-globalization relationship we find differs between landlocked and non-landlocked countries. Finally, considering the green growth agenda of the continent, regularization techniques can be employed to determine whether durable shared growth is driven largely by environmental factors or income growth and distributions.

## Footnotes

1 Green growth refers to achieving sustainable growth trajectories that is environmentally friendly (OECD, 2017).

2 Machine learning has gained attention in recent times due to its ability to detect relevant patterns in big data for prediction and analysis.

3 Countries in SSA have collectively signed onto the African Continental Free Trade Area.

4 Nomenclature of Territorial Units for Statistics.

5 Support-vector machine, neural network, lasso, boosted tree, regularized generalized linear model, and ridge.

6 Angola, Benin, Botswana, Burkina Faso, Burundi, Cabo Verde, Cameroon, Central African Republic, Chad, Comoros, Congo, Dem. Rep., Congo, Rep., Cote d'Ivoire, Ethiopia, Gabon, Gambia (The), Ghana, Guinea, Guinea-Bissau, Kenya, Lesotho, Liberia, Madagascar, Malawi, Mali, Mauritania, Mauritius, Mozambique, Namibia, Niger, Nigeria, Rwanda, Sao Tome and Principe, Senegal, Seychelles, Sierra Leone, South Africa, Sudan, Tanzania, Togo, Uganda, Zambia.

7 The KOF globalization index is a cross-country composite index comprising key dimensions of global interrelationships (i.e., economic, social, trade, financial, culture and political).

8 Inefficiency due to model complexity, specification problems, and/ or overfitting. Further, the traditional least squares estimator is not only less sparse but also more susceptible and sensitive to problems like multicollinearity and outliers.

9 Since the ordinary least squares technique and ridge regression cannot yield variable selection, their estimations are relaxed.

10 This adds to the regular $\lambda$ penalty.

11 In this study, we invoke the tenfold cross-validation.

12 Traditional estimation techniques such as the OLS cannot be employed either as the new variability introduced in the dataset by the regularization techniques are not captured by such techniques.

13 We include 56 out of the remaining 106 covariates as control against the backdrop that several alternative measures of globalization, institutional quality, and welfare are used.

14 List of instruments in POIVLR: transparency score, trade score, public management score, macroeconomic management score, gender equality score, financial sector management score, internet access (per 1 million of the population), mobile cellular subscription (per 100 of the population), fixed telephone subscription (per 100 of the population), fixed broadband subscription (per 100 of the population).

15 That is the distribution of economic growth in the training and testing sets.

16 See the Appendix.

17 For example, the case of the IMF and Ghana in 2015/2016 and the IMF and South Africa in 2021.
